# PI3-Kinase p110α Deficiency Modulates T Cell Homeostasis and Function and Attenuates Experimental Allergic Encephalitis in Mature Mice

**DOI:** 10.3390/ijms22168698

**Published:** 2021-08-13

**Authors:** José M. Rojo, María Montes-Casado, Laura Aragoneses-Fenoll, Gloria Ojeda, Umberto Dianzani, Pilar Portolés

**Affiliations:** 1 Departamento de Biomedicina Molecular, Centro de Investigaciones Biológicas Margarita Salas, CSIC, 28040 Madrid, Spain; 2 Unidad de Inmunología Celular, Centro Nacional de Microbiología, Instituto de Salud Carlos III, Majadahonda, 28220 Madrid, Spain; mmontes@isciii.es (M.M.-C.); laragoneses@isciii.es (L.A.-F.); gojeda@isciii.es (G.O.); 3 Interdisciplinary Research Center of Autoimmune Diseases (IRCAD), Department of Health Sciences, University of Piemonte Orientale (UPO), 28100 Novara, Italy; umberto.dianzani@med.uniupo.it; 4 Presidencia, Consejo Superior de Investigaciones Científicas (CSIC), 28006 Madrid, Spain

**Keywords:** phosphatidylinositol 3-kinases, CD4^+^ T-lymphocytes, CD4^+^ Treg, autoimmune experimental encephalomyelitis, multiple sclerosis

## Abstract

Class I phosphoinositide 3-kinases (PI3K) are involved in the development of normal and autoimmune responses, including Experimental Autoimmune Encephalomyelitis (EAE), a mouse model for human multiple sclerosis (MS). Here, the role of the ubiquitously expressed class IA PI3K p110α catalytic subunits in EAE has been analyzed using a model of Cre/flox mediated T cell specific deletion of p110α catalytic chain (p110αΔT). Comparison of two month-old (young) and six month-old (mature) p110αΔT mice and their wild type (WT) counterparts indicated loss of spleen CD4^+^ T cells that increased with age, indicating a role of p110α in their homeostasis. In contrast, CD4^+^ T regulatory (Treg) cells were enhanced in mature p110αΔT mice when compared to WT mice. Since Myelin Oligodendrocyte Glycoprotein (MOG) peptide-induced EAE is dependent on, or mediated by CD4^+^ T cells and CD4^+^ T cell-derived cytokines and controlled by Treg cells, development of EAE in young and mature WT or p110αΔT mice was analyzed. EAE clinical symptoms and disease scores in six month p110αΔT mice were significantly lower than those of mature WT, or young WT and p110αΔT mice. Furthermore, ex vivo antigen activation of lymph node cells from MOG immunized mature p110αΔT mice induced significantly lower levels of IFN-γ and IL-17A than young p110αΔT or young and mature WT mice. Other cytokines including IL-2, IL-10 or TNF-α showed no significant differences between p110αΔT and WT mature mice. Our data show a lower incidence of MOG-induced EAE in mature p110αΔT mice linked to altered T cell homeostasis and lower secretion of inflammatory cytokines.

## 1. Introduction

Experimental autoimmune encephalomyelitis (EAE) is a mouse model for human multiple sclerosis (MS), an inflammatory, progressively disabling disease in which elements of the immune system attack and destroy the myelin sheath covering central nervous system neurons [1]. Specific agents causing MS have not described, although it is known that different environmental and genetic factors significantly increase the risk to develop the disease [1,2]. The participation of an array of different cellular and molecular innate and adaptive immune elements at different sites and times during MS requires the identification of effective therapeutic targets, particularly in primary and secondary progressive MS [2]. In this regard, animal models of MS, such as EAE, are essential in current efforts to analyze disease mechanisms and new therapeutic approaches [3].

The development of EAE-induced by administration of Myelin Oligodendrocyte Glycoprotein (MOG) is dependent on CD4^+^ T cells producing Th1 and Th17 cytokines such as IFN-γ, TNF-α and IL-17A [3,4,5]. The severity of the disease is negatively controlled by regulatory cells including regulatory CD4^+^ T cells (Treg) [6,7,8] and anti-inflammatory cytokines such as IL-10 [6,9,10,11,12,13,14].

Class I phosphoinositide 3-kinases (PI3K) signals are involved in the development of normal and autoimmune responses, including the differentiation, proliferation, homeostasis, metabolism, effector mechanisms, and migration of immune cells (reviewed in [15,16,17,18,19,20,21,22,23]). Class I PI3K form heterodimers composed of regulatory and catalytic subunits that phosphorylate dually phosphorylated phosphoinositides of cell membranes (PI(4,5)P2) in the 3-OH position of the inositol ring to generate (PI(3,4,5)P3 (PIP3)). PI3Ks are activated by the recruitment of the regulatory subunits through membrane-proximal phosphorylated tyrosine motifs (in class IA PI3K) and G-protein receptor subunits (in class IB PI3K). Of interest to this work, Class IA PI3Ks participate in the differentiation of CD4^+^ T effector cells involved in EAE, including Th1/Th17 [24,25,26,27,28,29,30,31,32,33] and Treg cells [26,34,35,36,37,38,39].

The class IA regulatory subunits p85α, p50α, p55α, p85β and p55γ form dimers with p110α, p110β or p110δ catalytic subunits. Whereas the catalytic subunits p110α and p110β are ubiquitously expressed, the p110δ polypeptides are mainly expressed by hematopoietic cells [15,22,40]. Mouse CD4^+^ T lymphocytes and T cell lines express p110α and p110δ, with p110β isoforms being marginally expressed [41]. Many lines of evidence show the importance of p110δ in T cell differentiation and function, including the use of specific inhibitors, or the data from p110δ-deficient mice or humans, and from mice expressing p110δ kinase-dead mutants [24,25,26,28,29,30,31,32,33,34,35,36,37,38,39].

Unlike p110δ deficiency, p110α deficiency is lethal to embryo development [42], so the role of p110α in T cells needs to be explored using subunit-specific inhibitors or T-cell specific deletion or inactivation. Our previous data using different pharmacological inhibitors specific for p110α alone or in combination with p110δ show a role for p110α in different T- and B-dependent responses in vitro and in vivo, and the inhibition of EAE by a dual inhibitor of p110α and DNA protein kinase [30,41,43]. Intriguingly, we have recently shown that T-cell-specific deletion of p110α leads to enhanced activation of CD4^+^ and CD8^+^ T cell in vitro, including enhanced Th1 and Th17 responses that might be relevant to EAE development [31]. These data, plus the diminished proportion of CD4^+^ T lymphocytes observed in the absence of p110α [31] and the role of p110α in Treg function [39], prompted us to analyze the evolution of p110α-deficient T cells over time and its impact in the development of autoimmune encephalitis.

Here, we show that the loss of CD4^+^ T lymphocytes is maintained over time, but the proportion of Treg is enhanced in mice whose T cells lack p110α (p110αΔT mice). Besides, unlike two month old mice, mature, six month old p110αΔT mice develop a milder form of experimental encephalomyelitis. This suggests that subtle changes caused by p110α PI3K in the survival and function of T cell subpopulations during adulthood have a profound impact on the development of EAE, confirming the potential therapeutic interest of PI3K isoforms in MS.

## 2. Results

### 2.1. Altered Cell Populations in Secondary Lymphoid Organs from Mice with PI3-K p110α-Deficient T Cells

**Figure 1:**
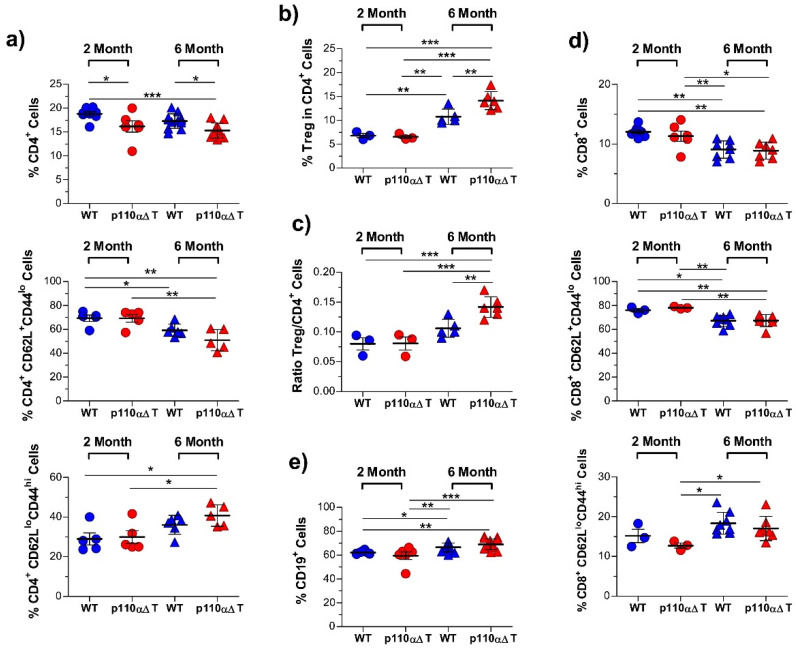
Flow cytometry analysis of spleen cell lymphocyte subpopulations in young, 2 month-old, or mature, 6 month-old wild type (WT) and p110αΔT mice. (**a**) Percentage of CD4^+^ T cells (top), naïve CD4^+^ T cells (CD62L^+^CD44^lo^, middle), and effector/memory CD4^+^ T cells (CD62L^lo^CD44^hi^, bottom) in young (circles), or mature mice (triangles). (**b**) Percentage of regulatory T cells (Treg, CD25^+^Foxp3^+^ in CD4^+^ T cells). (**c**) Ratio of Treg to conventional, non-Treg Foxp3^−^ CD4^+^ T cells. (**d**) Percentage of CD8^+^ T cells (top), naïve CD8^+^ T cells (CD62L^+^CD44^lo^, middle), and effector/memory CD8^+^ T cells (CD62L^lo^CD44^hi^, bottom). (**e**) Percentage of CD19^+^ cells (B cells). Data from individual WT (blue symbols) or p110αΔT mice (red symbols), as well as the mean ± SEM for each group, are depicted. Only significant differences between groups are indicated, as determined by one-way ANOVA (* *p* < 0.05, ** *p* < 0.01, *** *p* < 0.001). WT 2 month, *n* = 8 ((**a**) top, (**d**) top, (**e**)); *n* = 5 ((**a**) middle, (**a**) bottom); *n* = 3 ((**b**), (**c**), (**d**) middle, (**d**) bottom). p110αΔT 2 month, *n* = 6 ((**a**) top, (**d**) top, (**e**)); *n* = 5 ((**a**) middle, (**a**) bottom); *n* = 3 (**b**), (**c**), (**d**) middle, (**d**) bottom). WT 6 month, *n* = 12 (**a**) top, (**e**)); *n* = 8 (**d**) top, (**d**) middle, (**d**) bottom), *n* = 6 (**a**) middle, (**a**) bottom); *n* = 5 (**b**), (**c**)). p110αΔT 6 month, *n* = 11 (**a**) top, (**e**)); *n* = 7 (**d**) top, (**d**) middle, (**d**) bottom), *n* = 6 (**b**), (**c**)); *n* = 5 (**a**) middle, (**a**) bottom).

Our previous data indicated that the percentage of CD4^+^ T cells was significantly lower in the secondary lymphoid organs of young p110αΔT mice [31]. To assess whether this process was dependent on aging and its impact in distinct functional T cell subpopulations, spleen cell populations were compared in two and six month old female mice (Figure 1). In agreement with our previous data, the percentage of CD4^+^ T cells was significantly lower in p110αΔT mice than their age-matched wild type (WT) counterparts (Figure 1a); aging lowered the percentage of CD4^+^ T cells in p110αΔT or WT mice (Figure 1a). The fraction of CD4^+^ T cells with a naïve phenotype (CD62L^+^CD44^lo^) was significantly lower in six month-old than in two month-old p110αΔT mice (Figure 1a). This indicates a role for the p110α PI3-K subunit in the homeostasis of naïve CD4^+^ T cells. Besides, in agreement with published data, the proportion of CD62L^lo^CD44^hi^ effector/memory CD4^+^ T cells was enhanced in older mice; yet, no significant effect due to the mutant genotype was observed in same age mice (Figure 1a).

Figure 1b,c show that CD4^+^ Treg cells CD4^+^ were clearly higher in older mice, considering either the percentage of Treg cells in CD4^+^ T lymphocytes or the ratio of Treg to CD4^+^ T cells. Besides, the proportion of Treg cells was significantly enhanced in six month-old p110αΔT mice as compared to WT mice (Figure 1b,c). This might reflect the importance of the PI3-K p110δ subunit in Treg numbers and function [34,39], as p110δ is the main PI3-K subunit remaining in p110α-deficient T cells [31,41].

The analysis of whole CD8^+^ T cells or the naïve (CD62L^+^CD44^lo^) CD8^+^ population shows a significantly lower proportion in older mice but no significant differences between cells of p110αΔT and WT mice of the same age (Figure 1d, top and middle panels). In the same way as CD4^+^ T cells, CD62L^lo^CD44^hi^ effector/memory CD8^+^ T cells were enhanced in older mice, and no significant effect was observed between cells with or without p110α PI3-K from mice of the same age (Figure 1d, lower panel). The loss of T cells in older mice was compensated by an enhanced proportion of B cells (CD19^+^ cells (Figure 1e)) which was higher in p110αΔT mice.

### 2.2. Development of Experimental Allergic Encephalitis in Young and Mature p110αΔT Mice

One hallmark of naïve CD4^+^ T cells from p110αΔT mice is the enhanced secretion of certain cytokines such as IFN-γ when activated by anti-CD3 plus anti-CD28 antibodies [31]. This feature was maintained in cells from 6 month old mice, as shown in Figure S1. Besides, p110αΔT CD4^+^ Th1, Th17, or Tfh cells in vitro, or p110αΔT mice in vivo showed enhanced cytokine production including IL-17A, IFN-γ, or TNF-α [31].

Taking these data into account, plus the changes in naïve and Treg CD4^+^ T cells observed in p110αΔT mature mice, we analyzed the role of T cell p110α deletion in MOG-induced EAE by comparing the response in young (2 month old) and mature (6 month old) WT or p110αΔT mice. EAE was chosen as a model where: (a) development is dependent or mediated by effector CD4^+^ T cells and cytokines such as IL-17A, IFN-γ, TNF-α (reviewed in [4,5]); (b) Treg cells control the outcome of the disease [6,7]; (c) mature mice develop similar or stronger disease than young mice [44,45,46].

**Figure 2:**
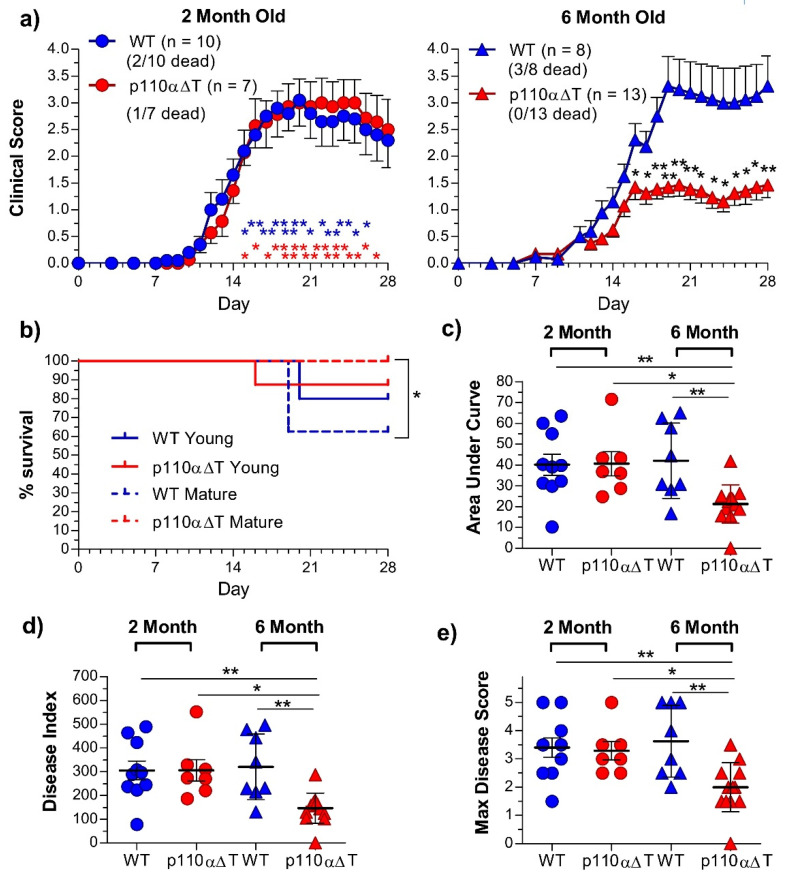
Comparison of Experimental Allergic Encephalomyelitis (EAE) disease parameters in young or mature wild type (WT) and p110αΔT mice, (**a**) Daily average Disease Score; (**b**) Percent Survival; (**c**) Area Under the Curve; (**d**) Disease Index; (**e**) Maximum Disease Score. In (**a**), the mean ± SEM of daily disease scores for each group is represented; deaths and the number of mice in each group are given in parentheses. The left graph in (**a**) shows data from young WT (blue circles) or p110αΔT mice (red circles); the right graph shows data from mature WT (blue triangles) or p110αΔT mice (red triangles). (**b**) Survival of young (solid lines) or mature (dotted lines) WT (blue lines), or p110αΔT mice (red lines) is shown. In (**c**–**e**), the data from individual young (circles) or mature (triangles) WT (blue symbols) or p110αΔT mice (red symbols), as well as the mean ± SEM for each group, are depicted. Data from two different experiments each of young and mature mice; individual data from young WT (*n* = 10, blue circles), young p110αΔT (*n* = 7, red circles), mature WT (*n* = 5, blue triangles), and mature p110αΔT mice (*n* = 8, red triangles) are shown. Only significant differences between groups are indicated, as determined by One-way ANOVA (**a**,**c**–**e**; * *p* < 0.05, ** *p* < 0.01) or the log rank (Mantel–Cox) test (**b**; * *p* < 0.05). In (**a**), asterisks in blue indicate significant differences between young WT and mature p110αΔT mice; red asterisks indicate significant differences between young and mature p110αΔT mice; black asterisks indicate significant differences between mature WT and p110αΔT mice.

Strikingly, EAE symptoms and disease scores in six month p110αΔT mice were clearly lower than those of mature WT, or young WT and p110αΔT mice (Figure 2). Significant differences between mature p110αΔT mice and all other experimental groups were observed in daily clinical scores, (Figure 2a). Furthermore, whereas no deaths were observed among the 6 month old p110αΔT mice, deaths occurred in all other groups, with significant survival differences between mature WT and p110αΔT mice (Figure 2a,b). Significant differences between mature p110αΔT mice and the other experimental groups were also observed concerning the areas under the curve (Figure 2c), the disease index (Figure 2d) and the maximal disease score (Figure 2e). The days of disease onset in the different groups (13.50 ± 0.73 (young WT), 13.71 ± 0.74 (young p110αΔT) 13.63 ± 0.75 (mature WT), and 15.80 ± 1.20 (mature p110αΔT)) were not significantly different.

Significant differences in the lymphoid populations from draining the lymph nodes of immunized mice at day 28 after EAE induction were mainly related to aging, particularly an enhanced percentage of Treg cells, enhanced Treg to Tconv ratios, or lower percentage of B lymphocytes (Figure S2). Yet, mature p110αΔT mice had a significantly lower proportion of CD4^+^ T lymphocytes than mature WT mice (Figure S2).

### 2.3. Cytokine Production by Lymph Node Cells from Young and Mature MOG-Immunized Mice

**Figure 3:**
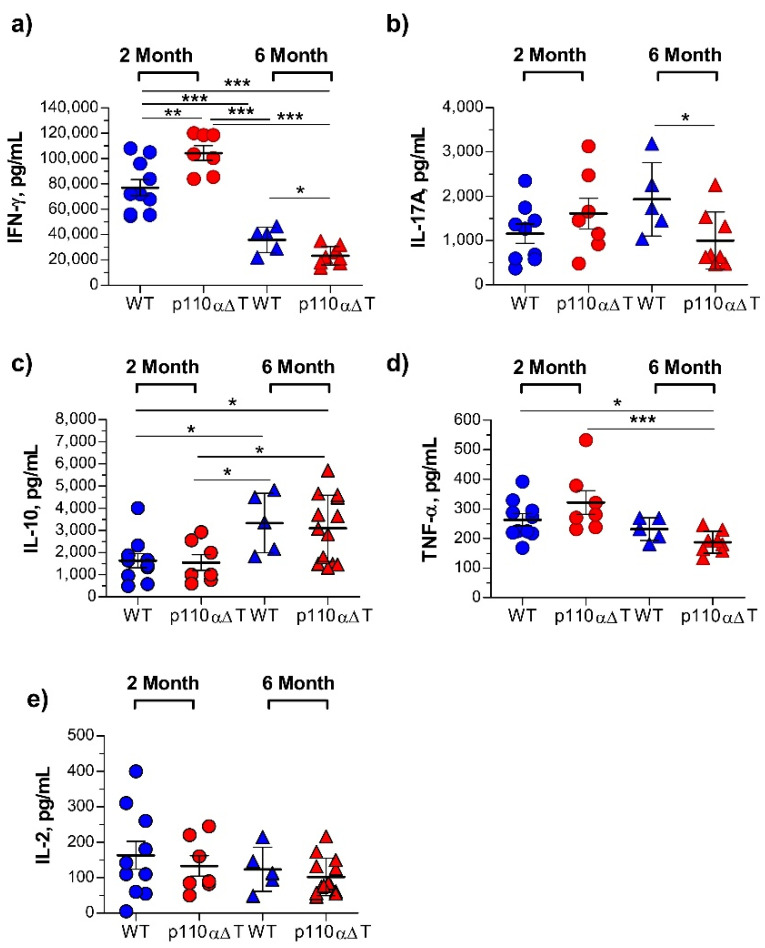
Cytokine secretion by lymph node cells from young and mature wild type (WT) or p110αΔT mice undergoing Experimental Allergic Encephalomyelitis (EAE). Lymph node cells from individual mice surviving on day 28 after Myelin Oligodendrocyte Glycoprotein (MOG) immunization were cultured for 96 h in the presence of MOG peptide antigen. Then, cytokine content in the supernatants was determined, as follows: (**a**) IFN-γ; (**b**) IL-17A; (**c**) IL-10; (**d**) TNF-α; (**e**) IL-2. Data from two different experiments each of young and mature mice; individual data from young WT (*n* = 10, blue circles), young p110αΔT (*n* = 7, red circles), mature WT (*n* = 5, blue triangles), and mature p110αΔT mice (*n* = 8, red triangles) are shown. Significant differences between groups are indicated, as determined by One-way ANOVA (* *p* < 0.05, ** *p* < 0.01, *** *p* < 0.001).

At 28 days of EAE induction, cells from the draining lymph nodes of the surviving mice were activated with the MOG antigen peptide, and cytokines in the supernatants were determined (Figure 3). Cells from young p110αΔT mice produced significantly higher levels of IFN-γ than cells from young WT mice (Figure 3a). The levels of IL-17A and TNF-α were also higher in young p110αΔT mice, although differences with young WT mice were not significant (Figure 3b,d). IL-10 levels were similar in both groups of young mice (Figure 3c). There were two major differences between young and mature WT mice concerning MOG-specific response, namely a drop in IFN-γ production and an enhanced IL-10 production by cells from older animals (Figure 3a,c). No significant differences were observed concerning IL-17A and TNF-α secretion between young and mature WT mice (Figure 3b,d).

Interestingly, unlike young p110αΔT mice, cells from mature p110αΔT mice undergoing EAE secreted significantly lower levels of IFN-γ than WT mice (Figure 3a). Furthermore, the levels of the inflammatory cytokine IL-17A were significantly lower in mature p110αΔT than in WT mice, and TNF-α was lower in mature p110αΔT mice when compared to either 2 month-old p110αΔT or WT mice (Figure 3b,d). IL-10 was enhanced in mature mice, but there were no differences between p110αΔT and WT of the same age. No significant differences were observed in IL-2 levels. These data, plus the significant correlation between clinical score and IL-17A, IFN-γ, but not IL-2 or IL-10 secretion (Figure S3), suggest that the lower incidence of EAE in p110αΔT mature mice is primarily due to their lower secretion of the IL-17A and IFN-γ inflammatory cytokines. Intriguingly, at the time considered there was an inverse correlation between TNF-α secretion and the EAE clinical score (Figure S3).

Whether some of the effects observed in mature p110αΔT mice were due to altered aging and/or exhaustion of conventional CD4^+^ T cells, or to the development and function of Treg cells was analyzed. So, the expression of markers linked to aging, exhaustion, and Treg function was determined. These included PD-1, ICOS, CXCR5, and Eomesodermin (Eomes) [47,48,49,50,51,52,53,54]. No significant differences between mature WT and p110αΔT mice were observed concerning PD-1, ICOS, CXCR5, or Eomes expression in CD4^+^ T lymphocytes, although there was a slight reduction of PD-1^+^ cells expressing ICOS (Figure S4a). Among Treg cells there were no significant differences in PD-1 expression; a minor reduction of ICOS^+^ or PD-1^+^ICOS^+^ Treg cells was appreciated (Figure S4b).

### 2.4. Early Anti-MOG Responses in Mature p110αΔT Mice

**Figure 4:**
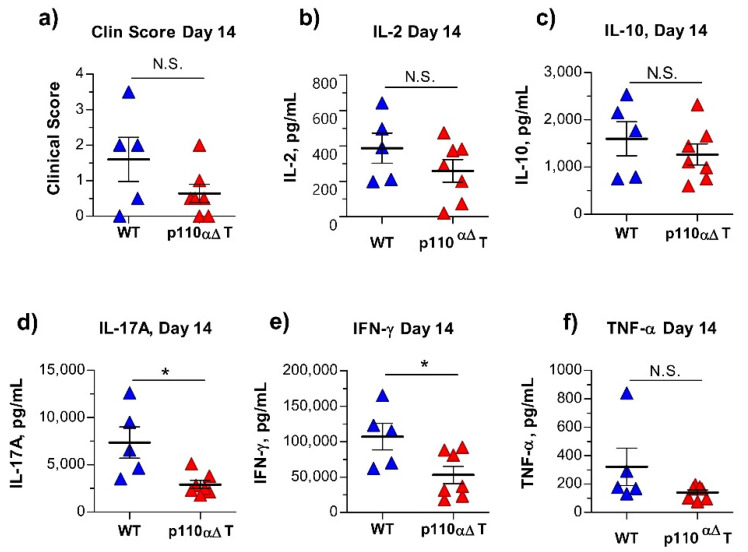
Secretion of cytokines by lymph node cells from individual mature wild type (WT) or p110αΔT mice obtained after 14 days of Experimental Allergic Encephalomyelitis (EAE) induction with Myelin Oligodendrocyte Glycoprotein (MOG) peptide. Cells were cultured for 96 h in the presence of MOG peptide antigen, and cytokine content in the supernatants was determined. (**a**) clinical EAE score; (**b**) IL-2; (**c**) IL-10; (**d**) IL-17A; (**e**) IFN-γ; (**f**) TNF-α. Data from mature WT (*n* = 5), and p110αΔT (*n* = 7) mice are shown. Significant differences between groups are indicated, as determined with the Mann–Whitney U test (**a**), or the unpaired two-tailed Student’ *t* test (**b**–**f**). * *p* < 0.05; N.S.: not significant.

To determine possible differences during EAE induction, the antigen-specific cytokine response was analyzed at an earlier time (day 14) where EAE symptoms could be detected, yet clinical scores between WT and p110αΔT mice were not significantly different (Figure 4a), and draining lymph node populations were similar (Figure S5). Cells from the draining lymph nodes were activated with antigen in vitro and cytokines in the supernatants determined. The data in Figure 4 show significant differences between p110αΔT and WT mice in IL-17A and IFN-γ contents, but not in secreted IL-2 or IL-10 (Figure 4b–e). Differences between p110αΔT and WT mice in TNF-α concentration were not significant (Figure 4f), although there was a significantly positive correlation between TNF-α secretion and EAE score at 14 days (Figure S6c).; significant correlations with EAE score were also found for IL-17A and IFN-γ (Figure S6a,b).

## 3. Discussion

We show here that mice whose T cells lack the PI3-K p110α subunit have a marked age-related difference concerning their susceptibility to EAE. Whereas the development of EAE symptoms in young p110αΔT mice is similar to that of WT mice, EAE is significantly milder in mature p110αΔT mice than in WT age-matched mice, or than in young WT and p110αΔT mice. A number of differences observed between mature WT and p110αΔT mice might contribute to the low susceptibility. First, the population of CD4^+^ T lymphocytes that is primarily involved in EAE development was significantly diminished in mature p110αΔT mice, yet the subpopulation of CD4^+^ Treg cells was clearly enhanced. Secondly, secretion of IL-17A and IFN-γ was significantly lower in cells from mature p110αΔT mice.

Since there is abundant literature showing the relevance of Treg cells, IL-17A and IFN-γ in the development and control of EAE [3,4,5,6,7,8,28], we suggest that differences in these factors are responsible for the distinct clinical outcome in mature p110αΔT mice.

First, we observed an enhanced proportion of Treg cells in mature WT mice that is further enhanced in mature p110αΔT mice. An enhanced proportion of Treg is likely contributing to EAE attenuation, and could be mediated by the activity of p110δ PI3-K, which is the main class IA PI3-K in CD4^+^ T cells remaining in the absence of p110α [31], and essential to Treg differentiation and function in mice and humans [26,34,35,36,37,38,39]. In contrast, Treg-specific deletion of p110α did not alter Treg proportions or function in the steady state, although it induced a slight reduction in EAE symptoms [39]. Indeed, we found no differences in Treg cells of young p110αΔT mice, whereas significant differences were observed in older animals. Recently, the attenuation of EAE in mice with PD-1-deficient Tregs has been linked to reduced PI3K-AKT signaling and the enhanced suppressive capacity of Treg cells [54]. However, we find no significant differences in PD-1 expression between Treg cells from mature WT and p110αΔT mice. Other recent data indicate that the loss of ICOS-mediated PI3K signaling is a key factor in enhancing Treg function [55]. In this regard, we and others have found lower ICOS PI3K signaling in p110α-deficient CD4^+^ T cells [41,56] or Treg cells [54]. This adds to the significantly lower expression of ICOS by the Treg cells from mature p110αΔT mice (Figure S4); all these factors together could contribute to enhanced Treg function in mature p110αΔT mice.

Surprisingly, p110αΔT mice have a lower percentage of CD4^+^ T cells [31], and our results show that this difference is maintained and enhanced in mature mice. A likely hypothesis is that the PI3-K p110α isoform is involved in the signaling needed for the survival of CD4^+^ T cells, as has been observed in B lymphocytes [57,58]. The loss of CD4^+^ T cells seems restricted to conventional, non-Treg CD4^+^ T cells, and might contribute to the lower incidence of EAE in mature p110αΔT mice. However, additional factors are needed, as EAE develops normally in young p110αΔT mice that also have a lower proportion of CD4^+^ T cells, and yet cells from these young mice produce higher amounts of IFN-γ and IL-17A when challenged in vitro, in agreement with our previous data [31].

Th1- and Th17-derived cytokines participate in the development of EAE and MS. Our data confirm their importance in the development of EAE, and in the differences between mature WT and p110αΔT mice. Thus, we found significant correlations between MOG-dependent secretion of IFN-γ and IL-17A and the EAE clinical score of mature mice. Furthermore, EAE attenuation in mature p110αΔT mice runs in parallel with significant reductions of both IFN-γ and IL-17A. The reductions were specific for these cytokines and the p110αΔT phenotype, and in the case of IFN-γ, took place in the context of a strong age-linked reduction of cytokine production. In addition, lower IFN-γ in mature p110αΔT mice was in contrast with the enhanced secretion in young mice, which was also found in isolated naïve CD4^+^ T cells from mature mice.

IL-10 secretion was also determined, as an anti-inflammatory cytokine that negatively controls EAE [6,9,10,11,12,13,14]. Antigen-dependent secretion of IL-10 was enhanced in mature as compared to young mice in the course of EAE, yet it was not different in WT or p110αΔT mice of the same age.

Th1 and Th17 cells are susceptible to Fas/FasL-induced cell death and IL-10-mediated suppression, respectively (reviewed in [5]). There is a possibility that in mature p110αΔT mice, Th1 cells express higher levels of Fas, or that Treg express enhanced levels of FasL, or both, so that Th1 responses are diminished. Similarly, the levels of IL-10Rα in Th17 and/or Treg cells from mature p110αΔT mice might be enhanced, rendering Th17 cells more susceptible to inhibition [59] and Treg more efficient at suppressing Th17 [60]; all these factors need to be addressed further.

Taken together, our data indicate that deletion of p110α in T cells induces the loss of certain CD4^+^ T cell subpopulations and favors the survival of Treg cells. Furthermore, in the absence of p110α there is enhanced production of important effector cytokines by activated or differentiated naïve cells (this manuscript, and [31]). Thus, in young WT mice the presence of p110α might tone down T cell responses, whereas the responses in mature WT animals might be favored by the improved survival of naïve T cells and a reduced population of Treg cells. Taking this into account, p110α inhibitors could be tested as a therapeutic mode of restraining excess responses in autoimmune diseases without compromising the development of antipathogen or anticancer adaptive responses.

## 4. Materials and Methods

### 4.1. Mice

C57BL/6J mice (WT), CD4-Cre (strain B6;D2-Tg(Cd4-cre) 1Cwi/CwiCnrm, [61]), and *Pik3caflox*, p110α^flox/flox^ [42] were bred in a C57BL/6J background from stock purchased from Charles River or the European Mouse Mutant Archive (EMMA, CD4-Cre) at the animal care facilities of the Centro de Investigaciones Biológicas Margaritas Salas (CSIC, Madrid, Spain), or the Instituto de Salud Carlos III (Majadahonda, Madrid, Spain) under specific pathogen free conditions. Mice referred to as young, two month old, or mature, six month old, were 8–10 weeks old or 24–28 weeks old, respectively, at the start of the procedures. Female mice with T cells deficient for PI3K p110α subunits (CD4-Cre^+/−^ p110α^flox/flox^; p110αΔT) and their Cre^−/−^ littermates (CD4-Cre^−/−^ p110α^flox/flox^; WT) were used. The experimental procedures were approved by the Ethics and Animal Welfare Committees of CSIC and Instituto de Salud Carlos III and were conducted according to institutional, national and European Union guidelines under project licenses PROEX 181/15 (to J.M.R., CSIC) and PROEX 330/15 (to P.P., ISCIII) issued by the Consejería de Medio Ambiente y Ordenación del Territorio, C.A. Madrid, Spain.

### 4.2. EAE Induction and Measurement

To induce EAE, mice were injected with 300 μg of a rat Myelin Oligodendrocyte Glycoprotein peptide (MOG_35–55_) in saline emulsified in CFA containing 500 μg of heat-killed *Mycobacterium tuberculosis*. The emulsion was administered s.c. at two sites in the upper and lower back (0.1 mL/site). Then, 0.5 μg of pertussis toxin (Calbiochem, Merck Life Science S.L.U. Madrid, Spain) dissolved in 0.1 mL of PBS were injected i.p. 2 h and 48 h after MOG administration. Mice were periodically analyzed for clinical symptoms of EAE that were graded with the following Disease Scores: (0) no clinical signs; (1) loss of tail tone; (2) wobbly gait; (3) hind limb paralysis; (4) hind and fore limb paralysis; (5) death—0.5 gradations were given to intermediate scores. The disease parameters used have been defined in detail previously [14,43,62]. These included daily average Disease Score in each experimental group; Day of Disease Onset (mean of the first day with clinical symptoms of mice in one experimental group); Maximum Disease Score (mean of the highest Disease Score in each of the mice of one experimental group), and Disease Index (daily disease scores in each of the mice were added, then divided by the average day of disease onset of the group multiplied by 100; the values were averaged for each group). The Area Under the Curve (AUC) was determined for the daily Disease Scores of each individual mouse with GraphPad Prism software v. 9.0.0 (GraphPad Software, San Diego, CA, USA); then, the individual values were averaged for each experimental group.

### 4.3. Antigen Activation Ex Vivo

At the completion of the EAE experiments, the draining axillar and inguinal lymph nodes from MOG-injected mice were excised, and single cell suspensions were obtained from individual mice. Then, the cells (1 mL at 10^6^ cells/mL in round-bottom tubes) were cultured for 96 h in Click’s medium supplemented with 10% heat-inactivated fetal bovine serum and 50 μg/mL gentamicin (culture medium) in the presence of 100 μg/mL antigen MOG peptide [43]. Culture supernatants were eventually taken to determine cytokine content.

### 4.4. CD4^+^ T Cell Isolation and Activation

Whole CD4^+^ T lymphocytes and naive CD4^+^ T were isolated from mouse spleen cell suspensions with the CD4^+^CD62L^+^ T cell isolation kit II (Miltenyi Biotec, Bergisch Gladbach, Germany). Whole CD4^+^ T lymphocytes obtained using this kit were devoid of CD4^+^CD25^+^ (Treg) T cells. Cells in culture medium (10^6^/mL; 1 mL/well) were activated in 24-well plates (Costar) pre-coated with anti-CD3 (YCD3-1, 5 µg/mL) in the presence of anti-CD28 antibodies (2.5 µg/mL), as described in detail in [31]. The supernatants were collected after 48 h of culture and the cytokines analyzed.

### 4.5. Cytokine Determination

Cytokines in culture supernatants (IL-2, IL-10, IL-17A, IFN-γ, and TNF-α) were quantified by capture ELISA using specific Ready-SET-Go! kits (eBioscience; San Diego, CA, USA) performed as specified by the manufacturer.

### 4.6. Antibodies

Rat anti-mouse CD3 antibody YCD3-1 [63] was obtained in-house by affinity chromatography from hybridoma supernatants; Syrian hamster anti-mouse CD28 (37.51) was purified from culture supernatants or obtained from BD Biosciences (San Jose, CA, USA). Antibodies coupled to different fluorochromes or biotin were purchased from eBioscience, ImmunoTools GmbH (Friesoythe, Germany), or BioLegend (San Diego, CA, USA), and included rat anti-mouse CD3, CD4, CD8, CD19, CD25, CD44, CD62L, CD185 (CXCR5), CD279 (PD-1), Foxp3 and Eomes; Armenian hamster anti-CD278 (ICOS), and adequate isotype controls.

### 4.7. Flow Cytometry

Single cell suspensions (0.5–1 × 10^6^) from spleen or spleen subpopulations, or from draining lymph nodes of mice undergoing EAE, were incubated in 50 µL of 10% heat-inactivated normal mouse serum in staining buffer (PBS, 0.05% NaN_3_, 5% heat-inactivated FBS) in the cold. Then, they were incubated with fluorochrome-labelled antibodies in the same buffer for a further 20 min in the cold. After washing, the stained cells were fixed with 1% paraformaldehyde in PBS and analyzed by flow cytometry; or fixed and permeabilized for intracellular staining using the Transcription Factor Staining Buffer Set (eBioscience). Staining with anti-Foxp3, anti-Eomes, or control isotype antibody was performed as indicated by the manufacturer. Eventually, flow cytometry data were acquired using a Beckman Coulter FC-500 flow cytometer (Beckman Coulter, Brea, CA, USA), or FACSCanto (BD Biosciences) or FACS LSR Fortessa (BD Biosciences) flow cytometers. Data were analyzed with FlowLogic (Inivai Technologies, Mentone, Australia), or FlowJo (Tree Star, Inc., Ashland, OR, USA, Version 10.0) softwares.

### 4.8. Statistics

Statistical analysis of data with the two-tailed Student’s *t* test, one-way ANOVA, the log rank (Mandel–Cox) test and the Pearson correlation coefficient, was performed with GraphPad Prism v. 9.0.0. Asterisks indicate significant differences (* *p* < 0.05, ** *p* < 0.01, *** *p* < 0.001). Scoring of clinical symptoms of EAE was performed in a blinded manner, without knowledge of genotypes.

## Data Availability

The data presented in this study are available on reasonable request from the corresponding authors.

## Supplementary Materials

The following are available online at https://www.mdpi.com/article/10.3390/ijms22168698/s1, Figure S1–Figure S6.
