# Supplementary material for: PI3-Kinase p110α Deficiency Modulates T Cell Homeostasis and Function and Attenuates Experimental Allergic Encephalitis in Mature Mice
Source: Int J Mol Sci. 2021 Aug 13;22(16):8698. doi: 10.3390/ijms22168698 (PMC8395417; doi:10.3390/ijms22168698)
Supplement: Supplementary file 1 [file ijms-22-08698-s001.zip › ijms-1321073-supplementary.pdf]

Ex vivo cytokine production by whole spleen CD4⁺ T lymphocytes or naive CD4⁺ T lymphocytes from mature 6 month-old mice

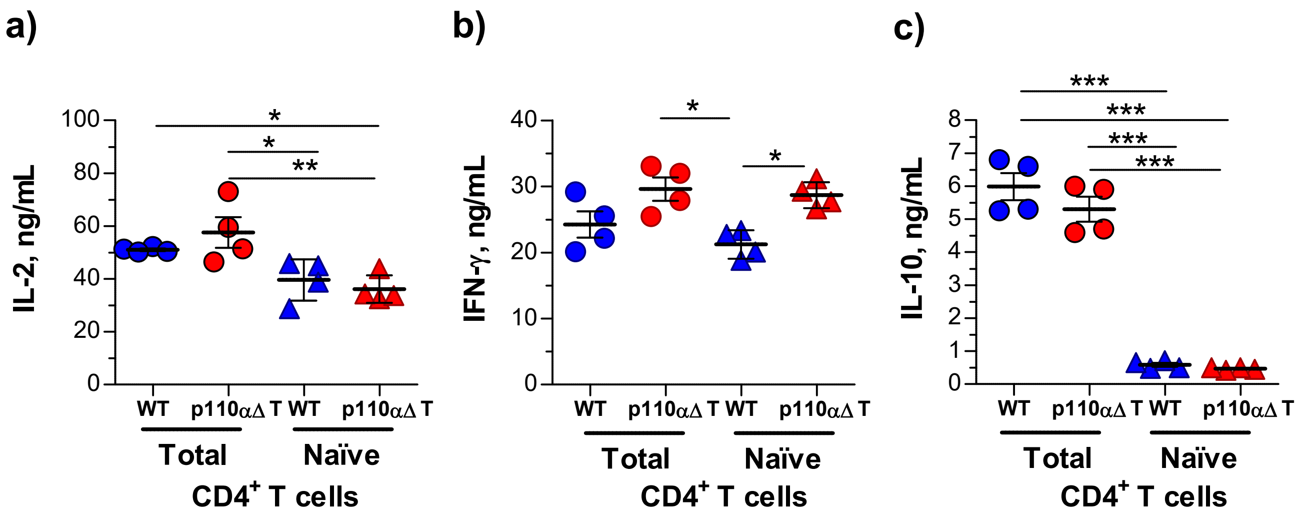

Figure S1.- Cytokine production by whole CD4⁺ T lymphocytes (Total, circles) or naive CD4⁺ T cells (triangles) isolated from the spleen of mature wild type (WT) (blue symbols), or mice with T cells deficient for PI3-K p110α subunits (p110αΔT mice, red symbols). Cells (10^6/mL) were activated in 24 well plates coated with anti-CD3 in the presence of anti-CD28 antibodies. At 48h the supernatants were taken and IL-2 (**a**)); IFN-γ (**b**)) or IL-10 (**c**)) was determined. Individual data from one representative experiment of two performed (n = 4/group) and the mean ± SEM for each group, are depicted. Significant differences between samples are shown, as determined by One-way ANOVA (*, p<0.05; **,p<0.01; ***, p<0.001).

Lymph node subpopulations in 2 month- or 6 month-old mice on day 28 after EAE induction

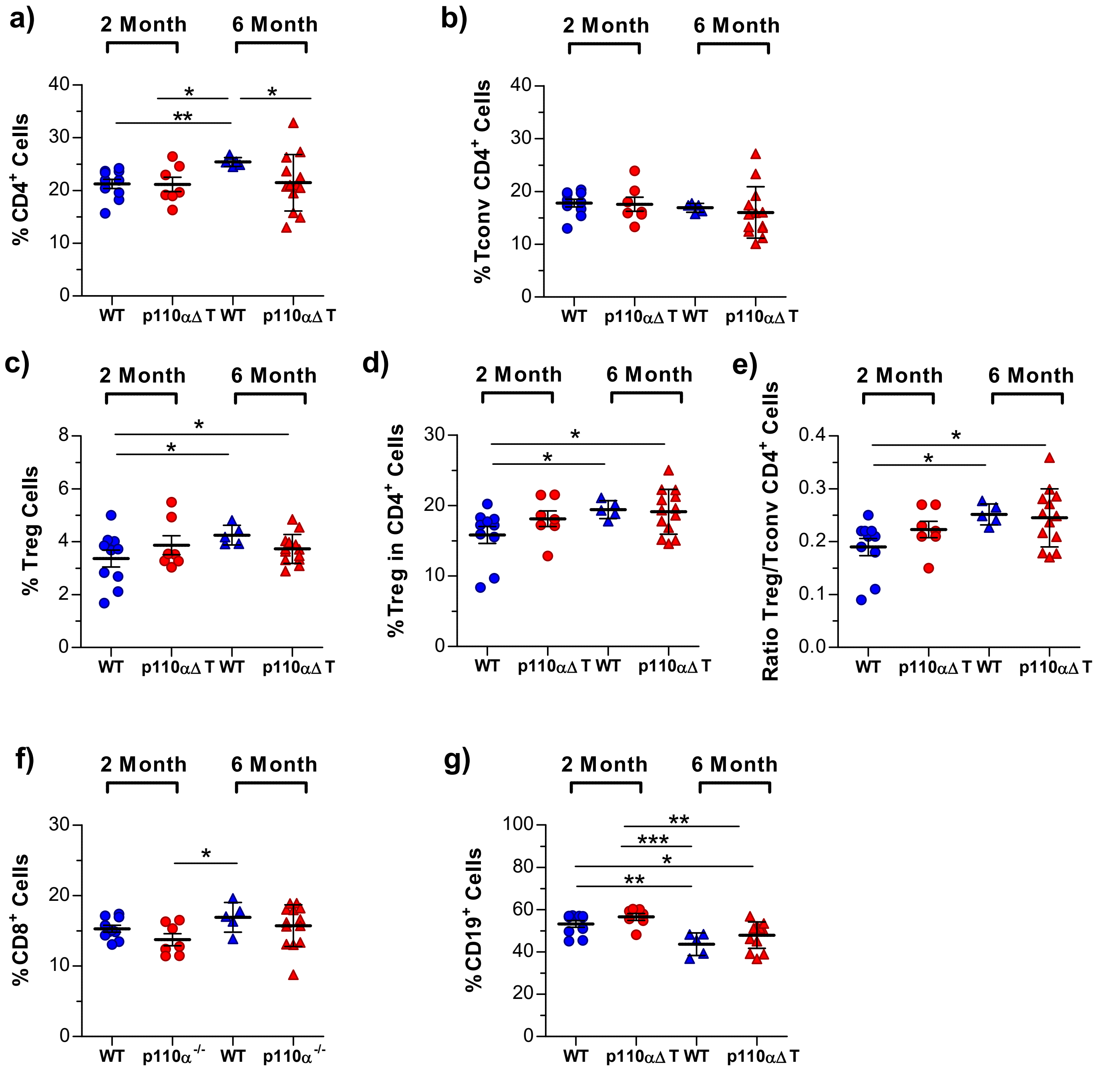

Figure S2.- Lymphocyte subpopulations in cells from draining lymph nodes of two-month and six-month old wild type (WT) and p110αΔT mice on day 28 after Experimental Allergic Encephalomyelitis (EAE) induction with Myelin Oligodendrocyte Glycoprotein (MOG) immunization. Percentage of (a) $CD4^+$ T cells; (b) conventional $CD4^+$ T cells (Tconv, $Foxp3^-CD4^+$ T); (c) regulatory T cells ($Foxp3^+$ $CD4^+$ T cells) in lymph node cells (d) regulatory T cells ($Foxp3^+$ $CD4^+$ T cells) in $CD4^+$ T cells. (e) Ratio of Treg to Tconv $CD4^+$ T cells. Percentage of (f) $CD8^+$ T lymphocytes and (g) $CD19^+$ B lymphocytes. Data from two different experiments each of young and mature mice. Individual data from young WT (n = 10, blue circles), young p110αΔT (n = 7, red circles), mature WT (n = 5, blue triangles), and mature p110αΔT mice (n = 8, red triangles) and the mean ± SEM for each group are shown. Significant differences between groups are indicated, as determined by One-way ANOVA (*, p<0.05, **, p<0.01).

EAE Clinical Score at day 28 of EAE vs Cytokine secretion in 6 month old mice

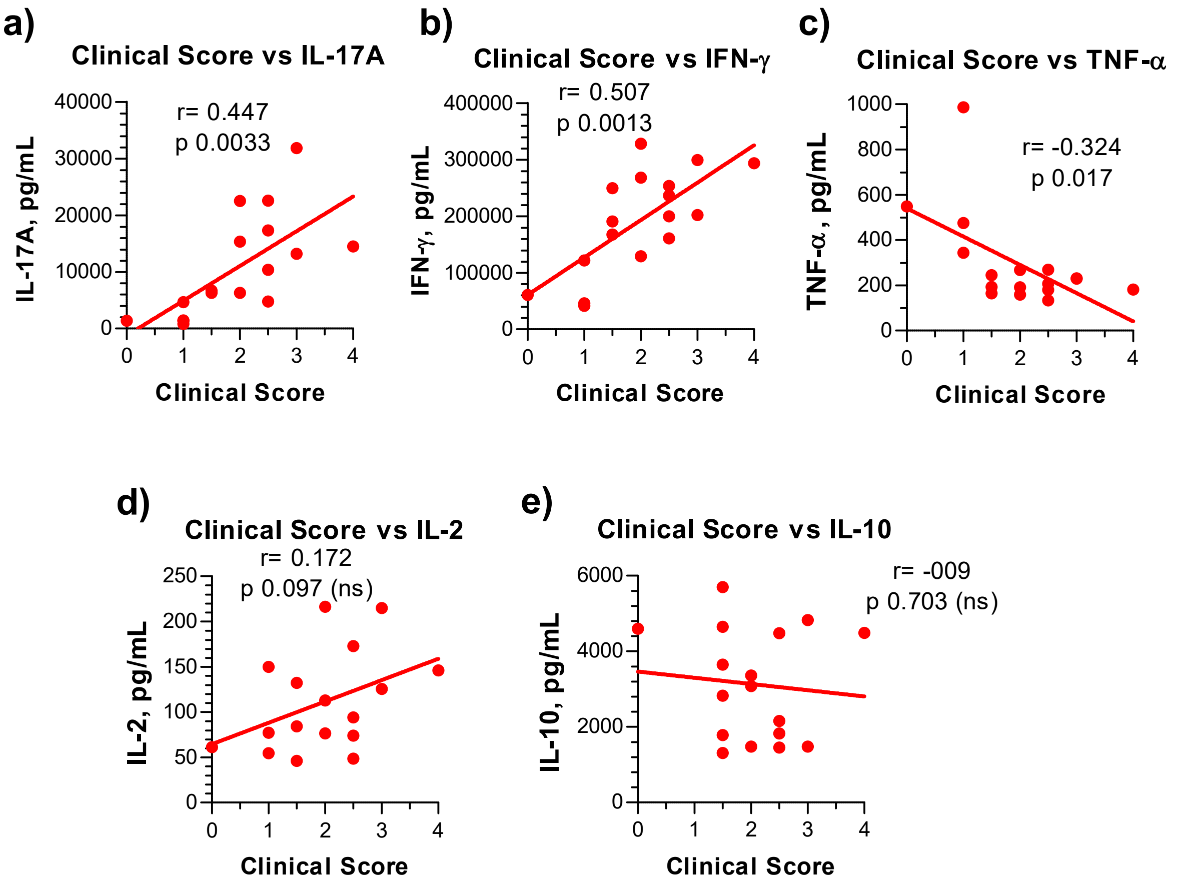

Figure S3.- Correlation between Experimental Allergic Encephalomyelitis (EAE) clinical scores and cytokine concentration in supernatants of lymph node cells from mature wild type (WT) or p110αΔT mice. Lymph node cells from individual mice surviving on day 28 after Myelin Oligodendrocyte Glycoprotein (MOG) immunization (n = 18) were cultured for 96h in the presence of MOG peptide antigen. EAE scores on day 28 were correlated with concentration of (a) IL-17A; (b) IFN-γ; (c) TNF-α; (d) IL-2; (e) IL-10 in supernatants. Values of p are indicated in the graphs; ns, not significant.

Expression of PD-1, ICOS, CXCR5, and Eomes in whole CD4⁺ T cells and CD4⁺ Treg cells from mature mice

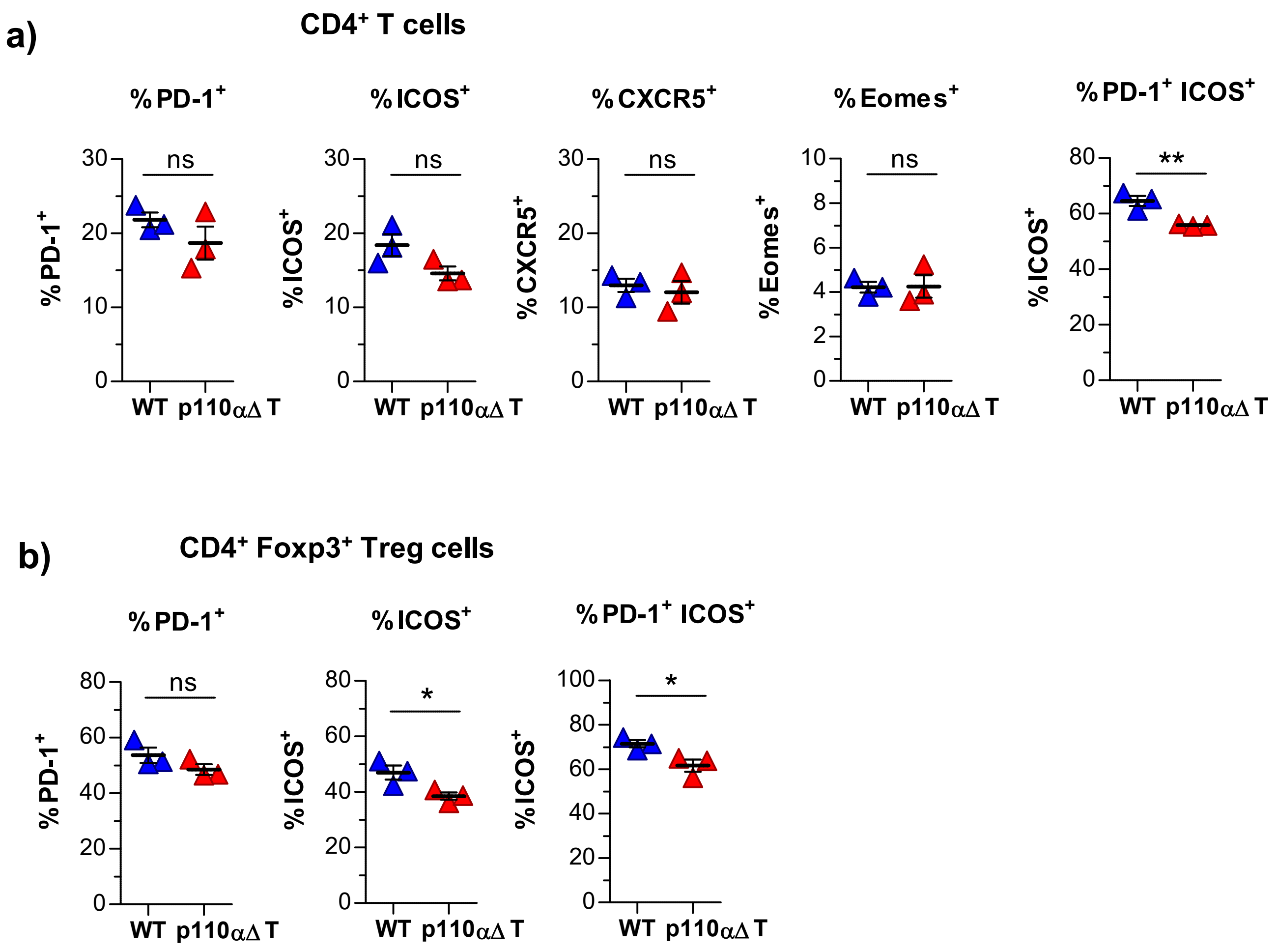

Figure S4.- Flow cytometry analysis of lymphocyte subpopulations in the spleen of six-month old wild type (WT) and p110αΔT mice. (a) The percentage of $CD4^+$ T cells positive for PD-1 or ICOS, CXCR5, and Eomes, or the percentage of $PD\text{-}1^+$ cells positive for ICOS, are shown, as indicated in the figure. (b) Percentage of regulatory T cells ($Foxp3^+$ $CD4^+$ T cells) expressing PD-1, or ICOS, and percentage of $PD\text{-}1^+$ cells positive for ICOS, as indicated. Data from individual WT (n = 3, blue symbols) or p110αΔT mice (n = 3, red symbols), and mean ± SEM for each group. Significant differences between groups are indicated, as determined with the unpaired two-tailed Student *t* test (*, $p<0.05$, **, $p<0.01$). ns, not significant.

Lymph node cell populations in WT and p110αΔT 6 month old mice on day 14 of EAE induction

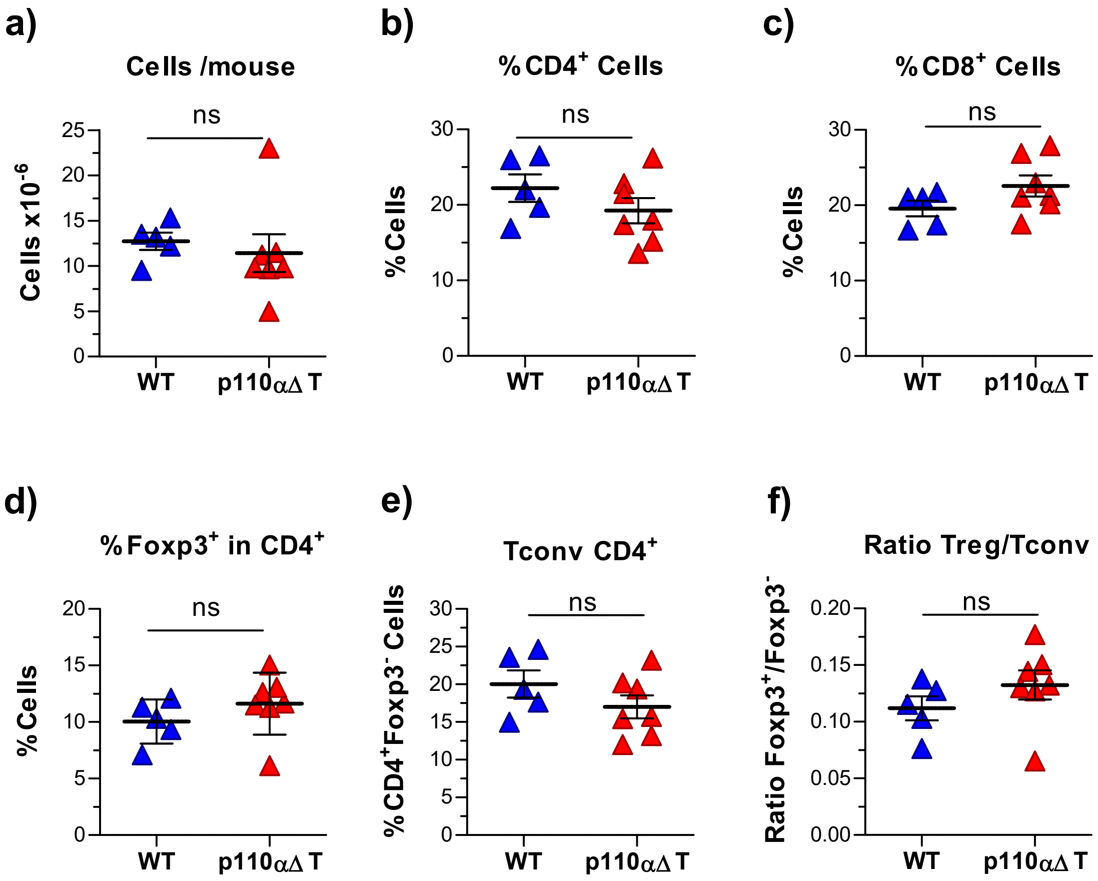

Figure S5.- Lymphocyte subpopulations in draining lymph nodes of six-month old wild type (WT) and p110αΔT mice on day 14 after Myelin Oligodendrocyte Glycoprotein (MOG) immunization. (a) Number of draining lymph node cells/mouse; (b) $CD4^+$ T cells; (c) $CD8^+$ T cells; (d) regulatory T cells ($Foxp3^+$ $CD4^+$ T cells) in $CD4^+$ T cells; (e) conventional $CD4^+$ T cells (Tconv, $Foxp3^-CD4^+$ T cells); (f) Ratio of Treg to Tconv $CD4^+$ T cells. Data from individual mature WT (n = 5, blue triangles) and p110αΔT mice (n = 7, red triangles), and mean ± SEM for each group are shown, ns, not significant, as determined with the unpaired two-tailed Student *t* test.

Clinical Score at day 14 of EAE vs Cytokine secretion in 6 month old mice

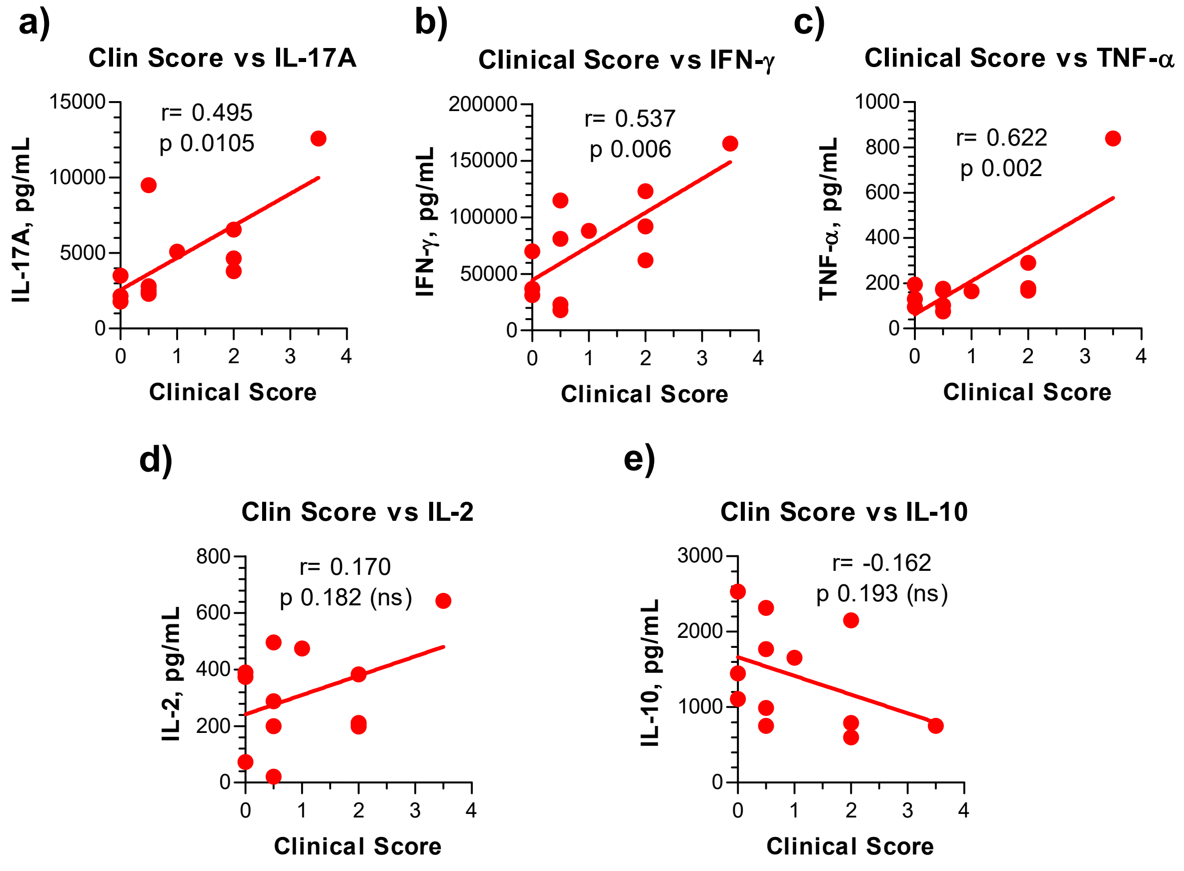

Figure S6.- Correlation between Experimental Allergic Encephalomyelitis (EAE) clinical scores and cytokine concentration in supernatants of lymph node cells from mature WT or p110αΔT mice on day 14 after Myelin Oligodendrocyte Glycoprotein (MOG) immunization. Lymph node cells from individual mice were cultured for 96h in the presence of MOG peptide antigen. EAE scores on day 14 were correlated with concentration of (a) IL-17A; (b) IFN-γ; (c) TNF-α; (d) IL-2; (e) IL-10 in the cultures. Data from mature mice are shown (n = 12). Values of p are indicated; ns, not significant.
